# Reusing Construction and Demolition Waste to Prepare Alkali-Activated Cement

**DOI:** 10.3390/ma15103437

**Published:** 2022-05-10

**Authors:** María V. Borrachero, Jordi Payá, Santiago Brito, Yasna Pamela Segura, Lourdes Soriano, Mauro M. Tashima, Jose María Monzó

**Affiliations:** 1 Institute of Concrete Science and Technology (ICITECH), Universitat Politècnica de València, 46022 València, Spain; jjpaya@cst.upv.es (J.P.); santiagobritopujols@gmail.com (S.B.); lousomar@upvnet.upv.es (L.S.); maumitta@upvnet.upv.es (M.M.T.); jmmonzo@cst.upv.es (J.M.M.); 2 Departmento Dengenieria de la Construction, Universidad de Magallanes, Av. Bulnes Nº 01855. C.P., Punta Arenas 6210427, Chile; yasna.segura@umag.cl

**Keywords:** valorisation, construction and demolition waste, blast furnace slag, compressive strength, microstructure

## Abstract

Large amounts of waste are derived not only from construction processes, but also the demolition of existing buildings. Such waste occupies large volumes in landfills, which makes its final disposal difficult and expensive. Reusing this waste type is generally limited to being employed as filler material or recycled aggregate in concrete, which limits its valorisation. The present work proposes reusing construction and demolition waste to manufacture alkali-activated cement to improve its sustainability and recovery. Construction and demolition waste (C&DW) from a demolition waste collection plant in Valencia (Spain) was physically and chemically characterised. This residue contained large fractions of concrete, mortar, bricks, and other ceramic materials. X-ray fluorescence (XRF) analysis showed that its chemical composition was mainly CaO, SiO_2_ and Al_2_O_3_. X-ray diffraction (XRD) analysis revealed that it presented some crystalline products, and quartz (SiO_2_) and calcite (CaCO_3_) were the main components. Blends of C&DW and blast furnace slag (BFS) were alkali-activated with mixtures of sodium hydroxide and sodium silicate. The corresponding pastes were characterised by techniques such as thermogravimetry and scanning electron microscopy (SEM). The alkali-activated mortars were prepared, and the resulting mortars’ compressive strength was determined, which was as high as 58 MPa with the 50% C&DW-50% BFS mixture. This work concluded that it is possible to make new sustainable binders by the alkali activation of C&DW-BFS without using Portland cement.

## 1. Introduction

The European Commission Waste Framework Directive provides basic waste management-related definitions. To comply with the objectives of this Directive, European Union Member States, by 2020, must use 70% non-hazardous waste from construction and demolition works [1]. Construction and demolition waste (C&DW) is assumed to comprise at least 30% of all total solid waste worldwide [2]. Depending on the construction type, several construction material waste types can be found in C&DW compositions: mortar, concrete, wood, bricks, ceramic sanitaryware, steel, etc.

In 2018, China produced 2360 million tonnes of C&DW. The EU generated a significant amount the same year: for example, Germany alone contributed 225 million tonnes [3]. China is the largest producer of C&DW, followed by India and the United States. To date, the main use of C&DW is to replace natural aggregates to produce mortar and concrete. Nevertheless, large-scale C&DW reuse towards a circular economy system needs to be enhanced [4].

In line with this, Lopez et al. [5] proposed action strategies based on five main stages: preconstruction; construction and renovation; collection and distribution; end of life; material recovery and production. Following the above strategies, four potential outputs for C&DW use were pointed out: substitution of 100% raw material for C&DW; recovered materials with partially recycled content to substitute materials of a similar nature; recovered material to substitute components of materials of a different nature; lowered energy demand.

Employing C&DW as a precursor in alkali-activated cements is a new way to valorise this waste material. The main difficulty of large-scale C&DW use is probably related to its high heterogeneity and, consequently, its low reproducibility. Indeed, most studies have focused on utilising specific C&DW parts/fractions. Ahmari et al. [6] studied using a mixture of ground waste concrete (GWC) and the fly ash (FA) types as precursors in geopolymeric pastes with different concentrations and proportions of NaOH and sodium silicate (SS). GWC was prepared with concrete made in the laboratory to analyse unconfined compressive strength (UCS), SEM/EDS, XRD and FTIR. The highest UCSs were obtained with the 50% GWC-50% FA mixture for all the NaOH concentrations (5 M and 10 M) and at two SS/NaOH ratios (1 and 2). According to the authors, the presence of calcium in GWC enhanced strength due to the co-existence of CSH gel and the incorporation of Ca^+2^ into the geopolymer structure.

Several studies have demonstrated the potential of ceramic waste as a precursor in alkali-activated cements. This material can be used alone or combined with other materials, such as blast furnace slag (BFS) or fluid catalytic cracking residue (FCC) [7,8,9]. An interesting research work was by Kioupis et al. [10], who proposed using rejected bricks as a precursor and glass waste to fabricate windowpanes as a component of the alkaline-activating solution. Glass waste was thermally treated with NaOH and water. The authors prepared different mixtures to obtain specimens with a maximum compressive strength of 32 MPa after 7 days of curing.

Vasquez et al. [11] utilised C&DW (concrete structure debris) to prepare single, hybrid and binary pastes with C&DW, cement (OPC) and metakaolin (MK), respectively. In the binary pastes, the authors considered the proportion between MK and C&DW to calculate the SiO_2_/Al_2_O_3_ molar ratio in the precursor. However, the molar ratio in the hybrid pastes was calculated only with the quantity of C&DW. In the single mixtures (only C&DW), SS addition enhanced the compressive strength of the sample activated with only NaOH, by 288%. The presence of 10% MK in the binary pastes yielded 46.4 MPa compressive strength at 28 curing days in an ambient temperature versus 25 MPa obtained from the paste with 100% C&DW. Finally, the compressive strength of the hybrid mixture with 30% OPC was 33 MPa, achieved under the same curing and activator conditions as the aforementioned pastes. The authors concluded that addition of MK and OPC enhanced the dissolution of several crystalline phases of C&DW and contributed to better mechanical properties.

Robayo–Salazar et al. proposed separating C&DW into fractions to prepare blocks fabricated with 100% waste. Fractions were concrete waste, mortar waste, masonry waste and ceramic waste [12]. The coarse aggregate was formed by concrete waste, and the fine aggregate was formed by mortar waste and ceramic waste (red and white). Finally, the precursor of the geopolymeric mixture was 10% OPC and 90% C&DW (25% concrete waste, 25% mortar waste, 25% masonry waste and 25% ceramic waste). The first part of the study involved performing the binder optimisation of the hybrid mixture (OPC-C&DW) at different NaOH + SS/precursor and NaOH/SS ratios. The optimal ratios were 0.35 and 0.34, respectively. The second part of the paper studied the evolution of a concrete’s compressive strength with 100% waste. The compressive strength of this concrete’s specimens was 42.9 MPa after 90 curing days and at ambient temperature. Finally, the solid concrete block preparation demonstrated this system’s suitability. The characteristics of these blocks met the specifications of a high-class structural block.

The same authors recently published a communication with an eco-house prototype [13]. They compared the behaviour of the alkali-activated blocks obtained from different precursors: natural volcanic pozzolan with ground granulated BFS, FA, C&DW and red clay brick waste. The only mixture that was considered as low-strength structural block was the mixture with C&DW because the block did not meet the water absorption criterion.

Two marine sediments and different C&DW fractions were studied as precursors to prepare pastes activated with KOH and SS [14]. Sediments substituted fractions of 10% and 30% tiles, bricks and concrete. The concrete paste proved to be the worst sample because this fraction had a small quantity of SiO_2_ and Al_2_O_3_. One of the studied sediments enhanced the compressive strength of all the C&DW fractions. The same conclusions were reached in another paper published by the same author, which demonstrated that concrete waste always yielded compressive strengths below 15 MPa versus the tile and brick waste, which, respectively, obtained 49.5 MPa and 57.8 MPa [15].

The present study aims to assess the potential of using real C&DW from a waste-sorting and recovery plant in Valencia (east Spain). Systematic waste characterisation was performed, and preliminary studies associated with its reactivity were carried out. The compressive strength and microstructural properties of the alkali-activated cements based on C&DW/BFS activated at two different SiO_2_/Na_2_O molar ratios were assessed for two different curing conditions. The use of BFS as part of the precursor is due to the fact that it is a well-known material in the literature of alkaline activation and that it has amply demonstrated its good behaviour both mechanically and in terms of durability [16].

The main objective of the investigation is to valorise C&DW residue in the construction industry. Its recovery would contribute to the fact that a waste produced by the same industry could be used again as a raw material, contributing to the development of the circular economy

## 2. Experimental Programme

### 2.1. C&DW Conditioning and Characterisation

**Figure 1:**
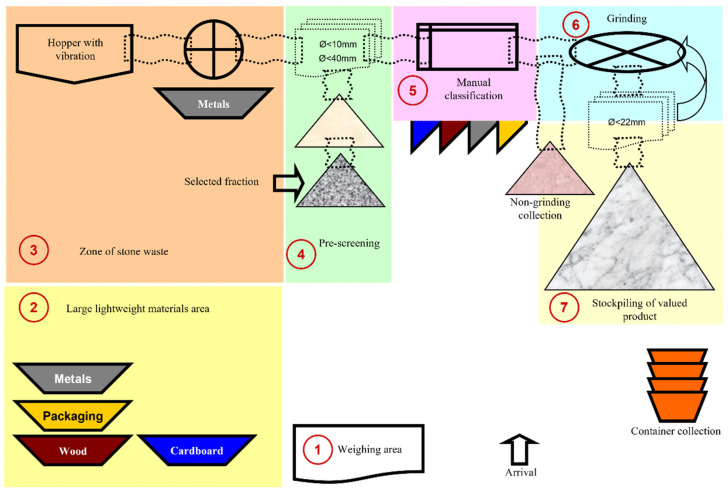
Diagram of the SECOPSA recovery plant.

Figure 1 shows a diagram of the different areas in the SECOPSA Medio Ambiente plant. This plant is located in Horno de Alcedo, Valencia (Spain).

**Figure 2:**
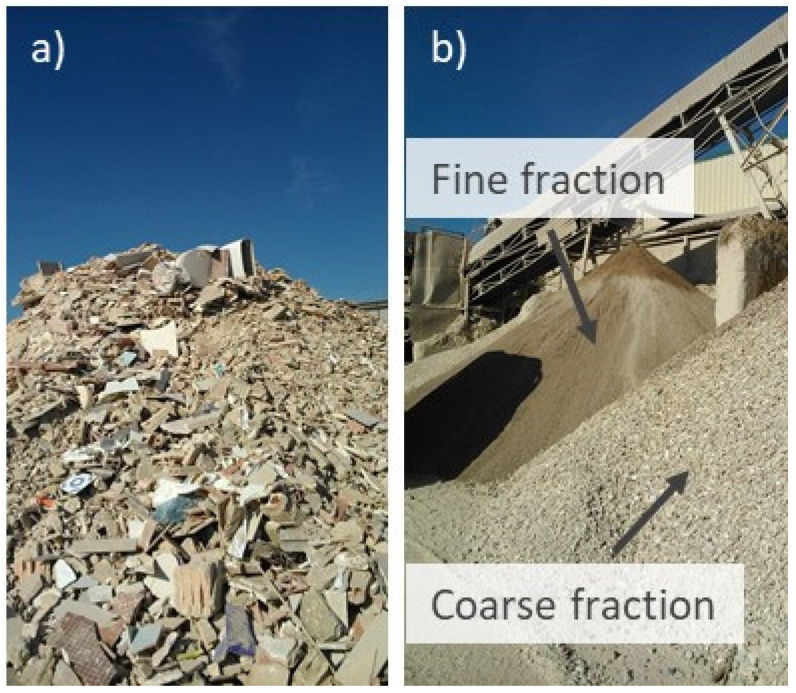
C&DW in the SECOPSA plant: (**a**) as-received material; (**b**) after the crushing/sieving process.

Figure 2a shows the as-received C&DW. Figure 2b illustrates the two fractions obtained in step 4 before manual classification.

Two C&DW fractions were obtained by sieving in step 4: fine and coarse fractions (see Figure 2b). In this study, the coarse fraction was selected to prepare alkali-activated cements because the finest fraction contained more impurities (plastic, wood chips, paper, gypsum). As this waste was of no value, it was subsequently taken to a landfill. The only valuable waste obtained in the plant was in step 7, which was later used as gravel.

About 500 kg of coarse C&DW was collected from the recovery plant and characterised for this study. C&DW was placed inside a jab crusher (Retsch BB20 model) to produce particles of size < 2 mm. This material was milled in a ball mill for 15 min (200 g of C&DW with 96 alumina balls in Gabrielli Mill 2 equipment) to obtain a similar mean diameter particle to the other binding materials used in this research. Different instrumental techniques were applied to characterise C&DW: X-ray fluorescence (Philips Magix Pro spectrometer); X-ray diffraction in a Brucker AXS D8 Advance of Billerica (XRD spectra were taken from 10° to 70° 2θ, at 20 mA and 40 kV, in an angle step of 0.02°); a laser dispersion granulometric analysis using Malvern Instruments Mastersize 2000 (measurements in aqueous medium); field emission scanning electron microscopy (Zeiss FESEM ULTRA 55); thermogravimetric analysis by Mettler Toledo TGA 850 (using alumina crucibles, and temperature range from 35 °C to 1000 °C at a heating rate of 20 °C/min in an air atmosphere). To analyse XRD pattern, DRXWin&Creafit 2.0 software was used. The samples analysed by microscopy were coated with carbon to make them conductive and to be observed in the equipment.

### 2.2. Other Materials

Calcium hydroxide (Ca(OH)_2_, 96% purity, supplied by Panreac Química SLU) was used to study the pozzolanic reactivity of C&DW following the evaluation method proposed by Tashima et al. [17].

BFS was supplied by Cementval SL (Puerto de Sagunto, Valencia, Spain). It was dried at 100 °C and dry-milled in a ball mill for 30 min (450 g of BFS with 98 alumina balls in Gabrielli Mill 2 equipment). The mean particle diameter obtained after milling was 26.09 µm. The main BFS compounds in the mass were: CaO (40.35%), SiO_2_ (30.04%) and Al_2_O_3_ (10.6%).

Siliceous sand, with a fineness modulus of 4.1 and a humidity percentage < 0.1%, was used to prepare the mortars. The alkaline solutions were composed of a mixture of commercial reagents: the mixture contained NaOH pellets (98% purity, Panreac Quimica SLU), Na_2_SiO_3_ (28% SiO_2_, 8% Na_2_O, 64% H_2_O, supplied by Merck S.L.U) and tap water.

### 2.3. Experimental Procedure

A preliminary study of the C&DW reactivity was performed to assess its potential use as a precursor in alkali-activated systems. First of all, the reactivity of C&DW with Ca(OH)_2_ (CH) was evaluated following the electrical conductivity method proposed by Tashima et al. [17]. The pH and electrical conductivity (using Micro PH2001 and Micro CM2201 supplied by Crison, respectively) for the C&DW and CH mixtures were monitored for the different CH:C&DW proportions (0.5:9.5; 1.0:9.0; 1.5:8.5; 2.0:8.0; 2.5:7.5 and 3.0:7.0) at 60 °C for 7 days. The suspension was prepared with 50 mL of deionised water in a 100 mL plastic screw-cap Erlenmeyer flask and the total amount of solid (C&DW plus CH) was set at 1 g. This flask was tightly sealed and placed inside a thermal bath (JULABO-SW22 equipment) with continuous shaking at 60 °C. Next, the corresponding amount of CH was added in each case to completely dissolve it, or until liquid saturation. At this point the electrical conductivity values were measured to obtain a control of the saturated suspension with CH, and C&DW was added. Electrical conductivity was measured every 24 h for 7 testing days.

An alkali-activated paste based on C&DW was prepared using 9 mol·kg^−1^ of sodium at the SiO_2_/Na_2_O molar ratio of 1.21 and a water/binder ratio of 0.45. This paste was cured for 7 days at 65 °C, and the thermogravimetric analysis and XRD pattern of paste were assessed.

Mortars were prepared using five different C&DW/BFS proportions (100/0, 80/20, 70/30, 60/40, 50/50 per mass) as a binder (precursor). For the activating solution, two different Na^+^ concentrations (7 and 9 mol·kg^−1^) were tested, for which two distinct SiO_2_/Na_2_O molar ratios (1.21 and 1.56) were assessed. The water/binder ratio and the siliceous sand/binder ratio were set at 0.45 and 3, respectively. Mechanical strength was measured on 40 mm × 40 mm × 160 mm specimens according to Standard UNE-EN 196-1 [18]. Compressive strength was measured after 3 and 7 curing days at 65 °C, and also after 28 and 90 curing days at room temperature. The selected alkali-activated pastes cured at 65 °C for 7 days (without sand) using similar mix proportions as the mortars were studied by FESEM.

**Figure 3:**
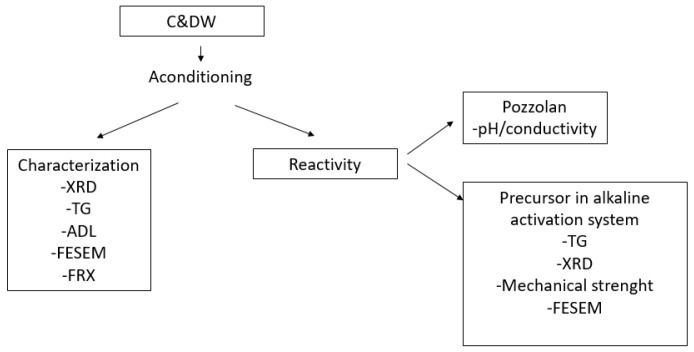
Scheme of the test carried out.

Figure 3 shows an outline of the studies carried out in the paper.

## 3. Results and Discussion

### 3.1. C&DW Characterisation

**Figure 4:**
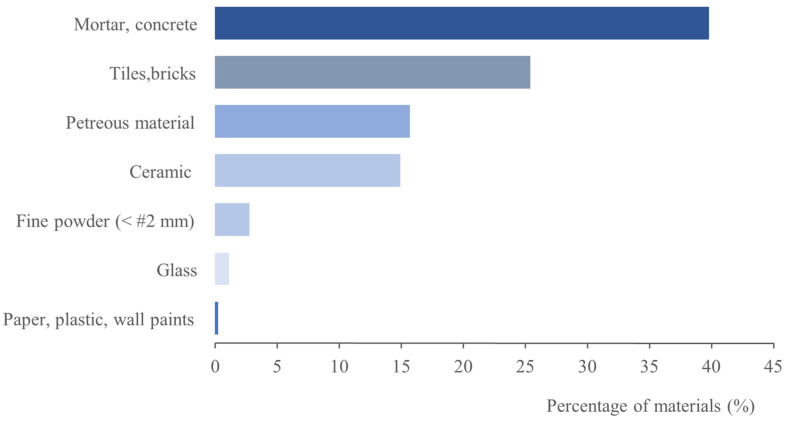
Percentage of the constituent materials in C&DW.

Figure 4 depicts the percentages of the construction material types found in C&DW. Low percentages of paper, plastic and wall paints were found (about 0.24%), whereas ceramic materials such as sanitaryware and ceramic floors appeared at 14.9%. The highest proportions were composed of mortar and concrete (approximately 39.8%) and tiles and bricks (about 25.4%). More than 65% C&DW was based on mortar, concrete, tiles and bricks. Petreous materials comprised mainly limestone materials, which are extremely abundant in the area where the plant is located.

**Figure 5:**
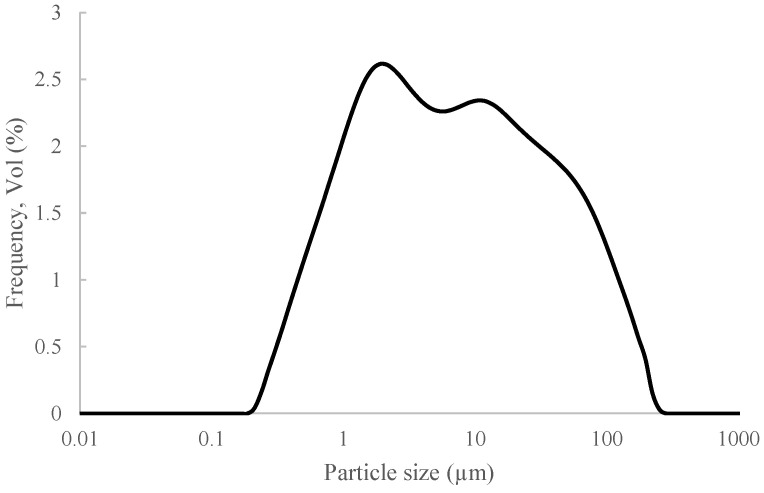
Granulometric distribution of the milled C&DW.

After the milling process, C&DW presented a wide granulometric distribution (Figure 5) and a mean particle diameter of 19.24 µm. The particle size distribution parameters for C&DW appeared, such as d (0.1), d (0.5) and d (0.9), which were 0.83 µm, 6.79 µm and 58.40 µm, respectively.

Table 1 shows the chemical composition of C&DW analysed by XRF. The main oxides present in waste were CaO (23.41%) and SiO_2_ (38.41%). These percentages were similar not only the C&DW investigated by Mucsi et al. [19] with CaO (21.1%) and SiO_2_ (38.7%), but also to the waste used by Robayo–Salazar et al. [12] with CaO (21.2%) and SiO_2_ (47.6%). Regarding the composition of the BFS, this residue had a lower percentage in CaO and slightly higher percentage in SiO_2_.

**Figure 6:**
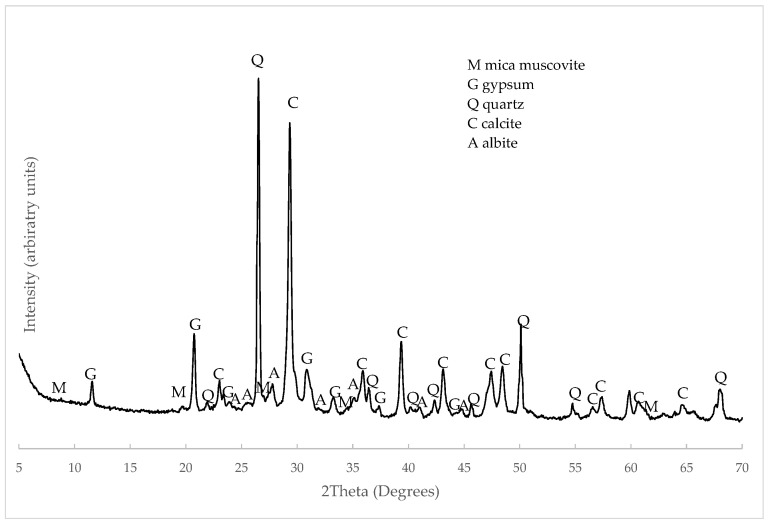
XRD pattern of C&DW.

The XRD pattern of C&DW appears in Figure 6. The main peaks corresponded to the presence of quartz (Q, SiO_2_, PDFcard 210816) and calcite (C, CaCO_3_, PDFcard 050583). Gypsum was identified as the secondary peak (G, CaSO_4_·2H_2_O, PDFcard 210816). These minerals derived from the composition of mortars and concretes (Portland cement and aggregates). Some peaks derived from the ceramic compounds present in C&DW, such as albite (A, NaAlSi_3_O_8_, PDFcard 090466) and muscovite mica (M, MgAlSi_4_O_10_(OH)_2_, PDFcard 210993). The presence of these crystalline compounds was similar to the C&DW studied by other authors [19,20].

**Figure 7:**
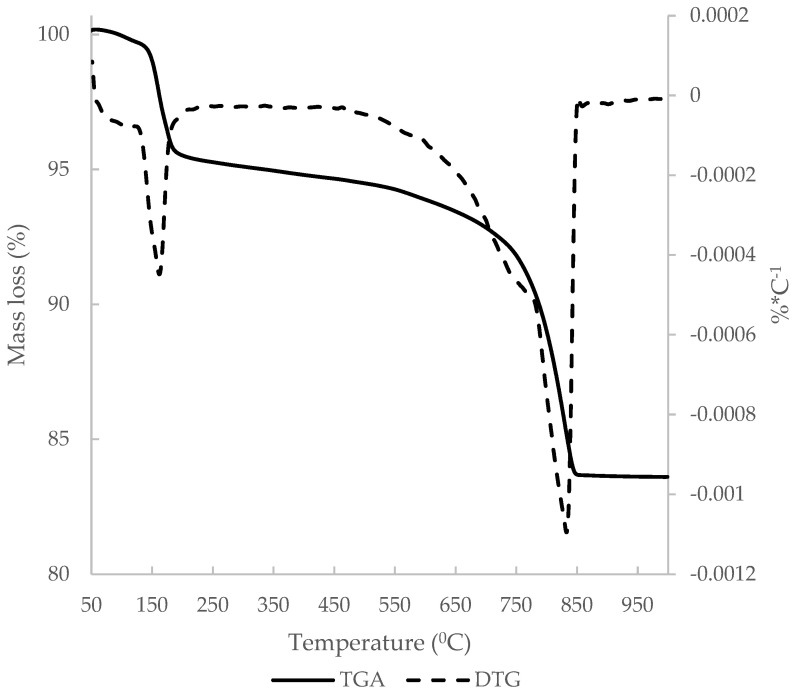
DTG curve of C&DW.

The derivative thermogravimetric curve (DTG) of C&DW is represented in Figure 7. The main peak occurred at around 850 °C, which was attributed to loss of CO_2_ from CaCO_3_. The other peak was around 150 °C and was attributed to the dehydration of gypsum and the hydrated compounds that derived from the hydration process of the Portland cement present in the mortar and concrete fractions. Total mass loss was 13.75% and the mass loss that derived from carbonates was 8.07%.

**Figure 8:**
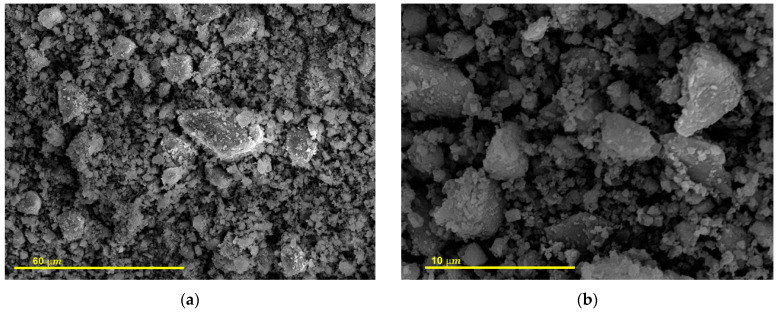
FESEM micrographs of the milled C&DW. (**a**) Magnification ×1000. (**b**) Magnification ×5000.

Figure 8 shows the FESEM images of the milled C&DW. The micrographs of this material displayed well-graded irregular-shaped particle distribution. Many particles were not porous.

### 3.2. C&DW Reactivity

#### 3.2.1. C&DW Reactivity as Pozzolanic Material

**Figure 9:**
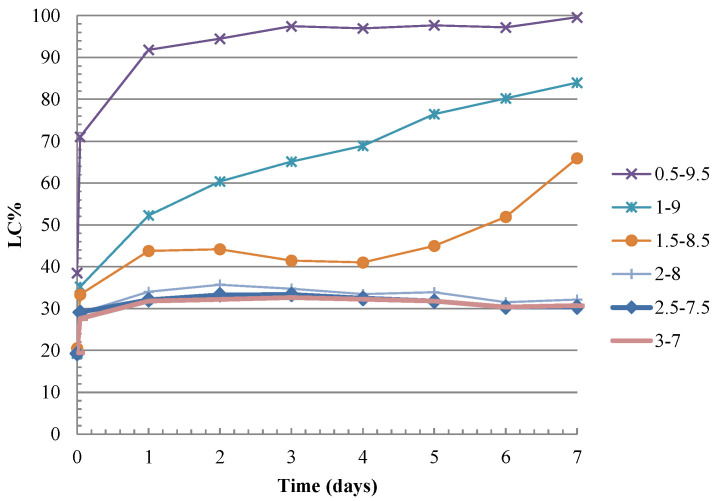
Loss of conductivity (LC%) versus time of the CH/C&DW mixtures.

Tashima et al. [17] proposed using a parameter denominated loss of electrical conductivity (LC%) to evaluate the unsaturation criterion of CH/pozzolan suspensions. A value higher than 30% for LC% would indicate that the aqueous solution was unsaturated in relation to CH and, therefore, pozzolan would have consumed the lime-forming cementitious products. As seen in Figure 9, only the suspensions with a small quantity of CH (0.5:9.5, 1.0:9.0 and 1.5:8.5) achieved the unsaturation setup for the CH:C&DW system.

According to the classification proposed by Tashima et al. [17], the pozzolanic reactivity shown by C&DW was low.

Frías et al. [21] used six different wastes generated during concrete waste crushing (particles of <5 mm). They assessed pozzolanic reactivity with an accelerated method in a pure pozzolan/CH system and concluded that these waste types exhibited medium–low fixation capacity. These results were consistent with the present research. Low reactivity was due to the high proportion in mortar, concrete and stones for C&DW, which are components with no pozzolanic material. Pozzolanic reactivity is attributed to the presence of ceramics and bricks.

#### 3.2.2. C&DW Reactivity as Precursor Material in Alkaline Activated Pastes

**Figure 10:**
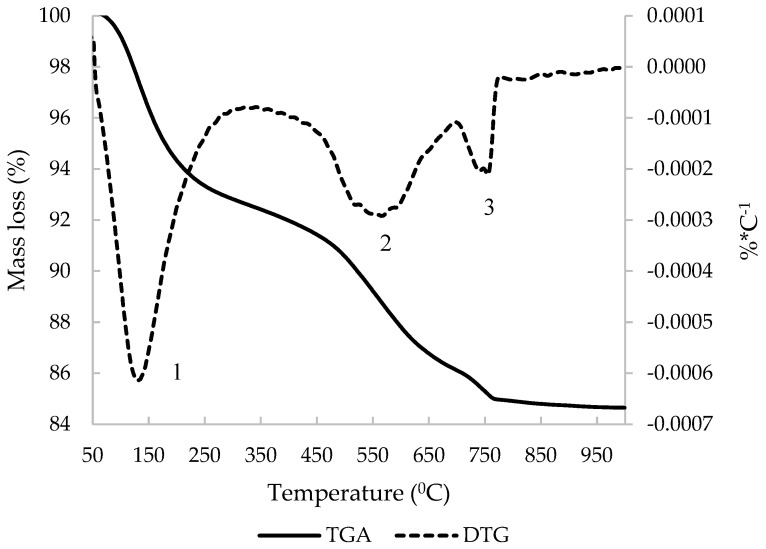
TGA and DTG curves of the alkali-activated paste of C&DW cured for 7 days at 65 °C.

A 100% C&DW paste was activated using sodium hydroxide and sodium silicate (9 mol·kg^−1^ of sodium, an SiO_2_/Na_2_O molar ratio of 1.21, and a water/binder ratio of 0.45). The paste was cured for 7 days at 65 °C to study the products that formed during the activation reaction. Figure 10 represents the TGA and DTG curves for this paste obtained within the heat interval 35 °C and 1000 °C. There are three significant zones which can be related to the decomposition of reaction products. In zone 1, mass loss can be attributed to the decomposition of CSH, CASH, NASH, and C(N)ASH that derive from either the alkaline activation reaction or unreacted waste. Similar products have been observed by Escalante et al. for active limestone with sodium hydroxide and waterglass [22]. The peak observed in Figure 7, centred at 850 °C, unfolded in two zones (zones 2 and 3). The peak in zone 3 appeared at the same temperature as that observed in the original waste (850 °C). A peak centred at 550 °C in zone 2. This phenomenon demonstrated that calcium carbonate reacts and produces calcium carboaluminate hydrate products. The formation of carboaluminates has been observed in binary and ternary pastes with cement, ground BFS, limestone filler and ground oyster shells [23]. The authors of those research works established that calcium carbonate was consumed to form calcium hemi- and monocarboaluminate phases. Other authors have established the interval between 300 °C and 650 °C for carboaluminates decomposition [24] or within 800–850 °C [25]. Payá et al. [26] observed carbonate decomposition at low temperature when calcium carbonate was activated.

**Figure 11:**
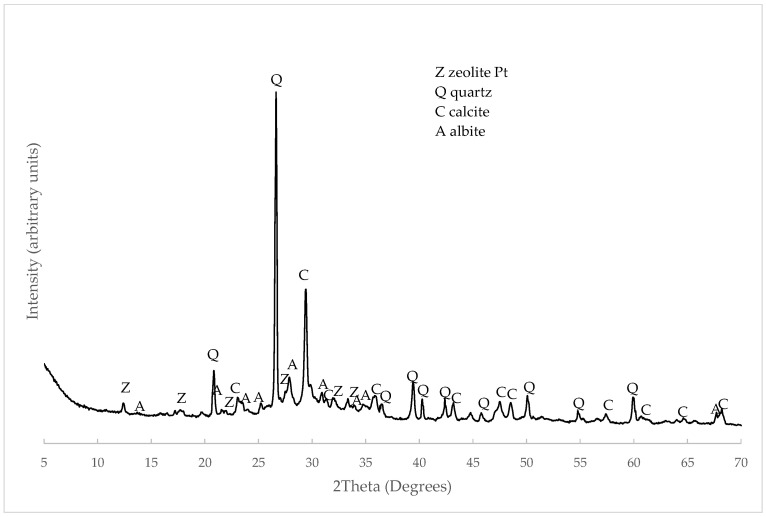
XRD pattern of the alkali-activated paste of C&DW cured for 7 days at 65 °C.

The presence of products derived from the alkali activation reaction was corroborated by the XRD analysis. Figure 11 represents the activated paste and the formation of zeolitic phases, specifically the formation of zeolite Pt (Z, Na_5.7_Al_5.7_Si_10.3_O_32_·12H_2_O, PDFcard 340524). The phases of the original material simultaneously appeared, such as albite, quartz or calcite.

### 3.3. Properties of the Alkali-Activated Mortars Based on the C&DW/BFS System

#### 3.3.1. Mechanical Strength

Although it was demonstrated that C&DW can be alkali-activated, the preliminary studies on the mortars demonstrated that the compressive strength of the mortar with only this waste type was very low. It was thought to use BFS as a precursor material along with C&DW. The use of BFS with other less reactive materials such as ceramic sanitaryware had been explored in previous research obtaining good results. In this research it was corroborated that the resistance of mortars with 100% BFS obtain compressive strength values higher than 90 MPa for 7 days of curing at 65 °C, and 80 MPa at 28 days of curing at room temperature. The author used a Na^+^ concentrations of 7.5 mol·kg^−1^ and an SiO_2_/Na_2_O molar ratios of 1.94 [27].

In the present experiment, the two selected SiO_2_/Na_2_O ratios (1.21 and 1.56) were chosen based on previous research [28], which employed hydrated cement. It was carbonated by two methods before being used as a precursor in an alkali-activated system. The prepared alkali-activated mortars contained this carbonated waste. The obtained compressive strength was between 10 MPa and 20 MPa at room temperature (28 curing days) depending on the carbonation conditions and the employed SiO_2_/Na_2_O ratio.

**Figure 12:**
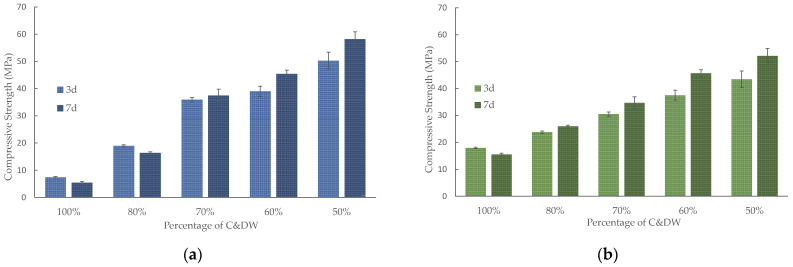
Compressive strength of the alkali-activated mortars cured at 65 °C at different SiO2/Na2O molar ratios: (**a**) 1.56; (**b**) 1.21.

The compressive strength for the C&DW/BFS mortars with different C&DW percentages cured at 65 °C are depicted in Figure 12. The compressive strength was 7.4 MPa after 3 curing days for the mortars activated at the 1.56 SiO_2_/Na_2_O molar ratio (Figure 12a) containing 100% C&DW. By replacing only 20% C&DW with BFS, a 201% increment in compressive strength was achieved. A linear increase in compressive strength took place with the rise in BFS and yielded up to 58.2 MPa after 7 curing days at 65 °C for the 50% sample.

Figure 12b presents the compressive strength of the mortars activated at the 1.21 SiO_2_/Na_2_O molar ratio. The maximum compressive strength (52.2 MPa) was yielded for the mortars containing 50% BFS after 7 curing days. A similar trend was generally observed for this series compared with the 1.56 SiO_2_/Na_2_O molar ratio. The main difference was related to the compressive strength of the 100% C&DW mortar. At the 1.21 SiO_2_/Na_2_O molar ratio, the 100% C&DW mortar yielded 18.0 MPa after 3 curing days, which was 2.5-fold higher than for the 1.56 SiO_2_/Na_2_O molar ratio. When the mixture contained 30% BFS or more, compressive strengths were slightly higher for the ratio with the smallest Na_2_O quantity. These results agreed with those published by Heikal et al. [29], who established that the compressive strength of BFS increases by alkali contents, but further increases can lower compressive strength. The increasing Na_2_O content probably resulted in changes in the products’ structure.

**Figure 13:**
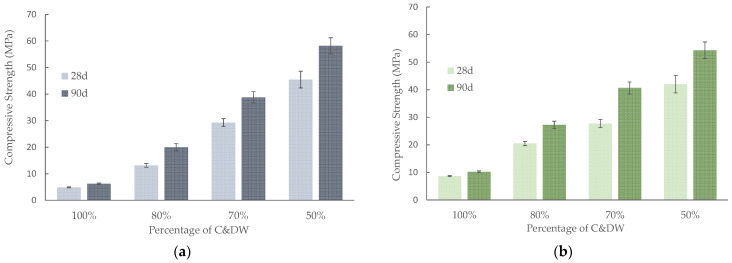
Compressive strength of the alkali-activated mortars cured at room temperature at different SiO_2_/Na_2_O molar ratios: (**a**) 1.56; (**b**) 1.21.

When the alkali-activated mortars were cured at room temperature (Figure 13), compressive strength slightly lowered, mainly for the 100% C&DW mortar compared with the results obtained at 65 °C. In this case, the mortar with the 1.21 SiO_2_/Na_2_O molar ratio yielded 10.3 MPa after 90 days, whereas compressive strength was 18.0 MPa after 3 curing days for the same mortar at 65 °C. This behaviour was observed only for the 1.21 SiO_2_/Na_2_O molar ratio. The mortars containing 70% C&DW presented very interesting mechanical behaviour, which yielded about 40 MPa after 90 curing days. The 50% C&DW mortars yielded more than 50 MPa compression.

The results obtained by adding C&DW, compared with a mortar with 100% BFS in other investigations, were lower, although the concentrations of the activators were not exactly the same. The contribution of C&DW to the alkaline activating reaction was not very high. Nevertheless, no deleterious effect was observed in the mechanical strength that developed when C&DW was added to the mortar. Thus, depending on the mortar application, up to 80% C&DW can be added to mortar to yield about 20 MPa depending on the SiO_2_/Na_2_O molar ratio. This fact can be considered a new sustainable way towards C&DW valorisation.

The improvement of mechanical resistance when using other precursors together with C&DW has already been studied by other authors such as Vasquez et al. [11] where the use of 10% MK or 30% OPC improved mechanical resistance by 85% and 33%, respectively, compared with a system with 100% C&DW.

#### 3.3.2. FESEM Studies

**Figure 14:**
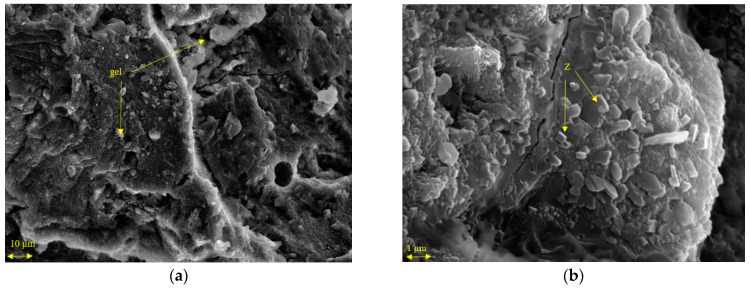

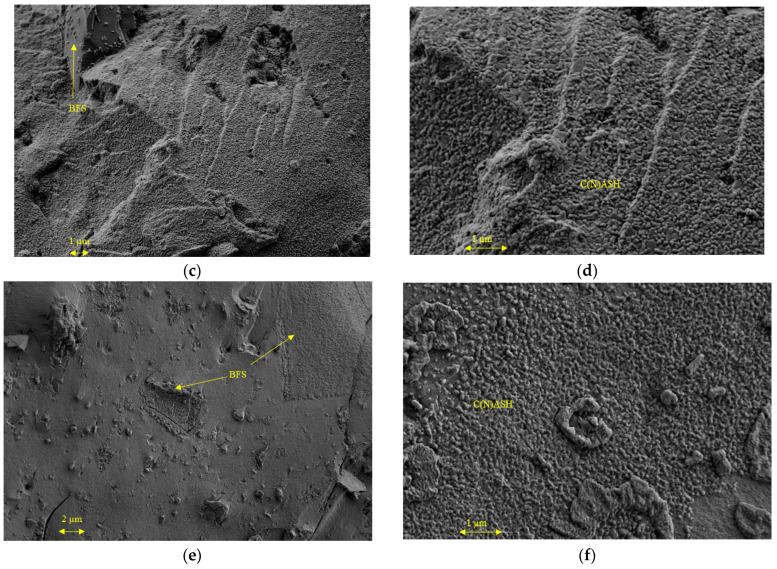
FESEM of the pastes activated at 65 °C for 7 days. (**a**) 100% C&DW. (**b**) 100% C&DW. (**c**) 70% C&DW–30% BFS. (**d**) 70% C&DW–30% BFS. (**e**) 50% C&DW–50% BFS. (**f**) 50% C&DW–50% BFS.

The activated pastes at the 1.21 SiO_2_/Na_2_O molar ratio and with different proportions of C&DW/BFS were studied by FESEM. Figure 14 depicts the micrographs for the pastes cured for 7 days at 65 °C.

The pastes activated with only C&DW (Figure 14a,b) presented a type of gel as the chemical reaction product. Gel morphology was similar to that previously reported during the activation of this waste type [20]. Villaquirán–Caicedo and Mejía de Gutiérrez [20] activated C&DW mixtures, which corresponded to zeolitic phases.

For the BFS-containing pastes (Figure 14c–f), the morphology of the products was more compact than those in the paste with only C&DW. The micrographs reveal particles of unreacted BFS and the principal product of the reaction was C(N)ASH gel. The formation of C(N)ASH gel in the pastes with BFS was reported by Moraes et al. when they activated BFS with sugar cane straw ash [30]. Yang et al. also indicated the presence of this gel while performing an EDS analysis on alkaline-activated BFS and coal gangue samples [31]. In pastes with BFS, the presence of zeolitic phases has not been observed.

## 4. Conclusions

The main conclusions drawn from the present research work are:

The studied C&DW contained considerable amounts of compounds CaO, SiO_2_, and Al_2_O_3_ to a lesser extent, which are very important for the alkaline activation process

The analysis of the electrical conductivity of the CH/C&DW suspensions showed that C&DW lacks important pozzolanic properties

The compressive strength results of the alkali-activated C&DW mortars using a mixture of NaOH and sodium silicate as an activator demonstrated the possibility of valorising this waste in this way, with up to 18 MPa after 3 days of curing at 65 °C. These resistances are perfectly valid for non-structural applications where resistance requirements are less important.

When BFS was added as part of the precursor, improvements in mechanical strength occurred, with values above 55 MPa after 7 days of curing at 65 °C. These improvements in mechanical strengths by adding BFS would allow mixed BFS/C&DW systems to be used in structural applications.

In view of the obtained results, one might think that these systems can be used as prefabricated elements of different characteristics. Depending on the necessary mechanical strength, the percentage of BFS to be used would vary. It also opens the possibility of valorisation of C&DW in binary mixtures with other precursors such as fly ash or the fluid catalytic cracking residue.

## Figures and Tables

**Table 1:** Chemical composition of the construction and demolition waste (C&DW) (wt %).

Al_2_O_3_	SiO_2_	CaO	Fe_2_O_3_	K_2_O	Na_2_O	MgO	SO_3_	Other	LOI *
8.43	38.76	23.41	3.67	1.71	0.22	1.29	3.80	1.24	17.46

* loss of ignition determined at 950 °C for 1 h.

## Data Availability

Data are contained within the article.
